# Idiopathic unilateral focal hyperhidrosis with social anxiety disorder

**DOI:** 10.4103/0019-5545.55094

**Published:** 2009

**Authors:** V. Anand Prakash Ghorpade

**Affiliations:** 1103, 24^th^ Main, J.P.Nagar I Phase, Bangalore, India

**Keywords:** Hyperhidrosis, focal hyperhidrosis, social anxiety disorder

## Abstract

Hyperhidrosis especially palmoplantar type are commonly referred to psychiatrist for management. Focal type of hyperhidrosis is less commonly seen more so associated with social anxiety disorder. Focal type is associated with variety of organic causes which has to be excluded before labeling it as Idiopathic variety. The variety of treatment that they are subjected to and its outcome are discussed in this case report.

## INTRODUCTION

Palmo-plantar hyperhidros is a well-known common social disabling disorder. Focal hyperhidrosis confined to exposed parts of the body is seldom seen in psychiatric practice and is likely to produce more disability in an impending social function say marriage. I am presenting here a case of idiopathic focal hyperhidrosis with social anxiety disorder, which was resistant to various known treatment modalities that naturally remitted.

A 31 yr old male, software engineer single, non-smoker, social drinker, planning to get married, presented with complaints of sweating only on his right forehead, shoulder, and forearm, since the age of 20 years. This symptom increased since 1 month while recovering from a viral fever episode. As the recovery progressed, sweating was found to be absent in familiar surroundings and increased in the company of strangers. Despite this, his work was satisfactory till 2004 when he observed increased sweating while traveling to office, when attending a call from a customer, or while attending to major problems at his office.

On his engagement day, on entering the hall, sweating increased which was very embarrassing to him. This gave rise to sleep disturbances and worry for which he sought consultation.

In the past history, he suffered from jaundice at the age of 12, CSOM at the age of 18 yrs without any residual defect. Dreams were mostly of anxious type in nature in the early years and more of depressing kind recently.

Pre-morbidly he is shy, introvert, worrying, God fearing, honest, with insecure feelings, and at times looses his temper.

On examination, he had divergent squint in the left eye. Central nervous system examination was completely normal.

When he consulted other specialists for this problem various investigations were carried out to rule out liver disorders (ultra sound of the abdomen), metabolic disorders, (diabetes, lipid disorders, electrolytes), heart disorders (stress test, echocardiogram), and thyroid disorders. M.R.I. of the brain and thoracic outlet did not reveal any space occupying lesions in brain and thoracic outlet.

Psychotherapy, relaxation exercises, minor tranquilizers, anti-depressants, and local botulin toxin injections could not allay his anxiety and associated sweating. Meanwhile, he was subjected to cervical ganglion block under local anesthesia, with which he had a temporary benefit. He reported that intensity of symptoms were tolerable after marriage as he was fully de-stressed with no medications.

Goldsmith[1] reported that focal hyperhidrosis could lead to embarrassing situations socially and psychological distress. The clinical profile of this case fits into the diagnosis of idiopathic unilateral circumscribed hyperhidrosis as described by Cunliffe *et al*.[2] and Kuritzcy *et al.*[3] The diagnosis of idiopathic focal hyperhidrosis was done, after ruling out variety of physical disorders such as brain infarction,[4,5] frontal lobe mengioma,[6] and cutaneous metastasis[7] which could give rise to focal hyperhidrosis. Davidson *et al*.[9] reported that hyperhidrosis is frequently seen in patients with social anxiety disorder (SAD) which supports the association of hyperhidrosis with SAD as is seen in the present case report. Hyperhidrosis in general and focal (exposed areas) hyperhidrosis in particular have to be managed on individual merits and demerits of available methods of treatment. Emotional component needs to be attended, instead of labeling them as “not a psychiatric disorder.”[8] The outcomes of treatment in such cases may be encouraging with a bio-psycho-social approach instead of a purely organic approach.
